# Phylodynamic analysis to inform prevention efforts in mixed HIV epidemics

**DOI:** 10.1093/ve/vex014

**Published:** 2017-07-28

**Authors:** Erik M. Volz, Nicaise Ndembi, Rebecca Nowak, Gustavo H. Kijak, John Idoko, Patrick Dakum, Walter Royal, Stefan Baral, Mark Dybul, William A. Blattner, Man Charurat

**Affiliations:** 1 Department of Infectious Disease Epidemiology, Imperial College, London, Norfolk Place W2 1PG, UK; 2 Institute of Human Virology Nigeria, Herbert Macaulay Way, Abuja, Nigeria; 3 Institute of Human Virology, University of Maryland School of Medicine, 725 W Lombard St, Baltimore, MD 21201, USA; 4 U.S. Military HIV Research Program/Henry M. Jackson Foundation for the Advancement of Military Medicine, Bethesda, MD, USA; 5 National Agency for Control of AIDS, Herbert Macaulay Way, Abuja, Nigeria; 6 Center for Public Health and Human Rights, Johns Hopkins University, Baltimore, MD 21218, USA; 7 Global Fund to Fight AIDS, Tuberculosis and Malaria, Chemin de Blandonnet 8, 1214 Vernier, Switzerland

**Keywords:** phylodynamics, HIV, coalescent

## Abstract

In HIV epidemics of Sub Saharan Africa, the utility of HIV prevention efforts focused on key populations at higher risk of HIV infection and transmission is unclear. We conducted a phylodynamic analysis of HIV-1 *pol* sequences from four different risk groups in Abuja, Nigeria to estimate transmission patterns between men who have sex with men (MSM) and a representative sample of newly enrolled treatment naive HIV clients without clearly recorded HIV acquisition risks. We develop a realistic dynamical infectious disease model which was fitted to time-scaled phylogenies for subtypes G and CRF02_AG using a structured-coalescent approach. We compare the infectious disease model and structured coalescent to commonly used genetic clustering methods. We estimate HIV incidence among MSM of 7.9% (95%CI, 7.0–10.4) per susceptible person-year, and the population attributable fraction of HIV transmissions from MSM to reproductive age females to be 9.1% (95%CI, 3.8–18.6), and from the reproductive age women to MSM as 0.2% (95%CI, 0.06–0.3). Applying these parameter estimates to evaluate a test-and-treat HIV strategy that target MSM reduces the total HIV infections averted by half with a 2.5-fold saving. These results suggest the importance of addressing the HIV treatment needs of MSM in addition to cost-effectiveness of specific scale-up of treatment for MSM in the context of the mixed HIV epidemic observed in Nigeria.

## 1. Introduction

In countries with hyper-endemic HIV epidemics (e.g. South Africa, Botswana, Lesotho, and Swaziland), the most efficient allocation of prevention activities may be to target all reproductive aged adults, often referred to as the general population in the context of HIV epidemiology. In most of West Africa, such as Nigeria, the opposite may be true. The majority of the HIV epidemics in this region are mixed (Lowndes et al. 2008), such that they are focused and propagated within the highest risk populations yet would be sustained if transmission in either population were interrupted (Boily et al. 2015). The study reported here employs phylodynamic analysis to more precisely inform the contribution of one key population (KP), men who have sex with men (MSM), to the larger Nigerian epidemic and to model the cost-effectiveness of targeted antiretroviral (ART)-based intervention.

It is difficult to ascertain HIV transmission patterns between different risk groups using observational data. Pathogen genomes from random samples of infected hosts provide one of the few sources of observational data that can inform precise estimates of transmission dynamics. Recent epidemiological history shapes the genetic diversity of rapidly evolving pathogens like HIV, which has spawned new approaches towards phylodynamic inference with the aim of harnessing pathogen genetic diversity to estimate epidemiological dynamics (Grenfell et al. 2004; Volz et al. 2013).

Most attempts to characterize HIV transmission using HIV sequence data have focused on identifying closely related subsets of sequences or ‘clusters’ in high income countries with high quality molecular surveillance due to routine resistance testing (Lewis et al. 2008; Wertheim et al. 2014; Ratmann et al. 2016). Such clustering may indicate an epidemiological relationship, however, rates of clustering are known to be confounded by incomplete sampling, loss to follow-up, and variables correlated with recency of infection (Volz et al. 2012; Poon 2016). In HIV epidemics of sub-Saharan Africa, sequence samples comprise a small proportion of the large number of total infections, and thus a random sample of sequences will yield few close epidemiological relationships(Volz and Frost 2013). Even when a sample contains no transmission pairs, a wealth of epidemiological information may be inferred from HIV sequence data, since changes in transmission and migration of lineages over long time scales influence HIV genetic diversity and phylogenetic patterns(Rosenberg and Nordborg 2002). In situations with low sampling density, model-based phylodynamic inference methods have become standard. Such modeling approaches have also been useful for estimating the early growth rate of the HIV epidemics (Kretzschmar et al. 2013) and tracing the origins of HIV in central Africa (Faria et al. 2014).

Here, we utilized HIV *pol* sequence data from several populations in Nigeria, a West African country with the second highest absolute HIV burden in the world (UNGASS 2014), to inform our understanding of the phylodynamics of HIV transmission and the potential bridging of infection between concentrated epidemic among MSM and a representative sample of newly enrolled treatment-naïve HIV-positive reproductive-aged adults (i.e. the general population). This report characterizes transmission patterns within the MSM population in Abuja, Nigeria and transmission between risk groups. In addition, HIV test-and-treat as a prevention strategy and its cost-effectiveness in disrupting HIV transmission dynamics is investigated.

## 2. Methods

### 2.1 Study populations

The Abuja-based *TRUST Cohort*, an ongoing longitudinal implementation research study of MSM recruited through respondent driven sampling (RDS) has an HIV prevalence observed to be approximately 10 times higher than among all reproductive aged adults (Baral et al. 2015; Charurat et al. 2015). The cohort has high rates of multiple sexually transmitted infections, a high incidence of HIV, and a high rate of bisexuality with 56% of cohort members reporting having female sexual partners in the last 12 months and with 28% of MSM living with HIV reporting having concurrent regular male and female partners (Supplementary Fig. S1).

The *TRUST* Cohort consisted of 806 MSM clients enrolled between March 2013 and December 2014 in a community-based trusted venue in Abuja. At the time of this analysis, the HIV population prevalence in MSM was 45% (10 times that of the general population) and HIV incidence rate was 13.9 per 100 person-years (95%CI, 4.7–33.3) based on detection of incident infections in the longitudinal cohort follow-up. The purpose of the study was to engage and retain MSM in HIV care, treatment, and prevention services and to gain an understanding of social and behavioral approaches that prevent HIV transmission. The median age of the participants was 23 years (interquartile range [IQR], 7). Sixty-six percentage of HIV-infected participants were not on ART at the time of enrollment and the median CD4 count was 318 cells (IQR, 137).

The *NeuroAIDS* Cohort (Akolo et al. 2014) consisted of 77 HIV-infected men and 139 HIV-infected women enrolled between April 2011 and September 2013 from two tertiary facilities in Abuja (University of Abuja Teaching Hospital [UATH] and National Hospital [NHA]). The purpose of the study was to characterize prevalence and incidence of neurocognitive impairment among treatment naïve HIV-infected adults enrolled in HIV care and treatment programs. Individuals were enrolled in an unselected fashion (i.e. not selected a priori because of suspected neurocognitive impairment). Over 90% were ART naïve at enrollment with a median CD4 count of 335 cells (IQR 249) and median log viral load of 4.5 (IQR 1.2). Around 84% were asymptomatic (WHO stage 1). The median age of the participants was 34 years (IQR 11), with 52% married and 15% unemployed (77% female).

The *TDF* Cohort was a retrospective cross-sectional study of archived samples from 175 HIV-infected patients enrolled in HIV treatment and care program between November 2006 and December 2007 and who had virologic failure. The purpose of the study was to determine HIV drug resistance patterns among patients who initiated first-line ART. The mean age was 38.1 (standard deviation [SD], 8.5) and 49% of the participants were female. The median CD4 cell count was 128 cells/μL (IQR, 169) and the median log viral load was 4.7 (IQR, 1.3).

The *ACTION* Cohort is one of the largest PEPFAR-supported programs in Nigeria. Viral load testing was conducted for patients to confirm HIV treatment failure. A total of 191 samples from patients who resided in Abuja between 2011 and 2014 underwent genotypic analysis for drug resistance. The median age was 36 (IQR, 13) and 58% of the patients were female. The median CD4 cell count was 154.

### 2.2 Pol sequencing

Plasma viruses with viral load (VL) > 1000 copies/ml were sequenced at the Institute of Human Virology Nigeria, Abuja, Nigeria, using established in-house methods developed and optimized for Nigerian isolates. Characterizing the genetics of 659 HIV-positive subjects was done using RNA extracted from plasma. Detailed sequencing protocols and quality control are described in the Supplementary Material.

### 2.3 Phylogenetic analysis

**Figure 1.**
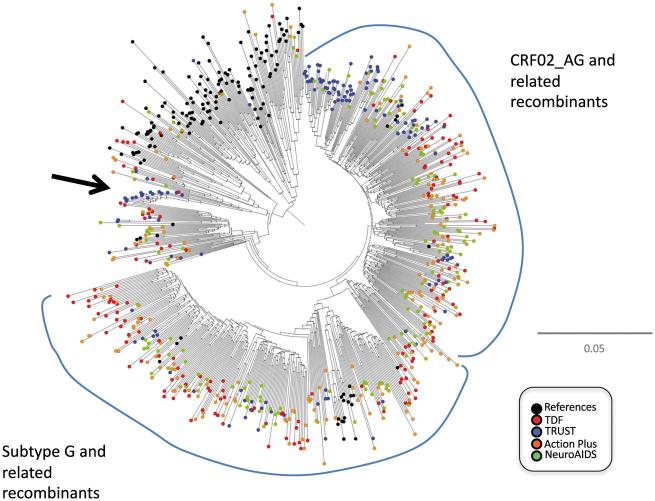
Neighbor-joining phylogeny of HIV-1 *pol* sequences from 659 individuals in Abuja, Nigeria. The arrow depicts a cluster of related sequences belonging to the subtype A1 radiation.

Assignment of subtype or circulating recombinant form (CRF) was performed combining tree-phylogenies, sliding-window BLAST, and jumping profile Hidden Markov Model (Schultz et al. 2009). A nucleotide alignment containing subtype reference sequences from the Los Alamos HIV Sequence Database (http://www.hiv.lanl.gov/) was assembled. The overall genetic diversity in the data set was assessed by nucleotide sequence analysis using the Kimura 2-parameter/neighbor-joining method implemented in MEGA (Tamura et al. 2011). Descriptive statistics were obtained using JMP version 10 in SAS. A neighbor-joining phylogeny and subtype assignment for sequences from the different data sources are illustrated in Fig. 1 and Supplementary Fig. S2.

A molecular clock analysis was used to produce dated phylogenies (with calendar time to common ancestry) using BEAST 1.8 (Drummond and Rambaut, 2007). Codons where primary antiretroviral drug resistance mutations were detected (i.e. codons 30, 32, 33, 46, 47, 48, 50, 54, 76, 82, 84, 88 and 90 in protease, and codons 41, 65, 67, 69, 70, 74, 100, 101, 103, 106, 115, 138, 151, 181, 184, 188, 190, 210, 215, 219 and 230 in RT) were first removed from the alignments. Separate phylogenetic analyses were conducted for sequences of subtypes CRF02_AG and G which together comprised the majority of sequences. We used a GTR substitution model with discrete gamma heterogeneity of rates between sites, and a relaxed uncorrelated molecular clock and a Bayesian skyline demographic prior. BEAST Markov chains were run for 300 million iterations. Only data from the TRUST and the NeuroAIDS studies were used for the molecular clock analysis and the phylodynamic analysis. For subsequent phylodynamic analysis, a random sample of 20 trees was drawn from the posterior distributions for subtypes G and CRF02_AG and then combined with a common ancestor in the distant past (code to perform this operation is available at https://github.com/emvolz/nigeria_hiv_model/misc).

### 2.4 Epidemiological modeling

Epidemiological dynamics were based on standard mathematical models that have previously been fitted to non-genetic surveillance data (Granich et al. 2009; Eaton et al. 2012). The structured coalescent models account for incomplete sampling and differential sampling rates of MSM and the general population. Transmission rates vary depending on stage of infection, sex, risk group, diagnosis status, and treatment status. Patients were classified into five stages of infection based on CD4 including a one-year early HIV infection period (EHI). Stage of infection of each patient at the time of sampling was based on recent testing history (last negative date) and CD4 count. The models account for secular trends in the force of infection, which has generally decreased through time due to behavioral change and increased diagnosis and treatment. The models account for change in diagnosis and treatment rates through time, and separate effects of diagnosis and treatment on reducing transmission rates and on improving longevity. Models of HIV epidemiological dynamics were developed using systems of ordinary differential equations (ODEs) with compartments corresponding to different risk groups, stages of infection, diagnosis and treatment status, and different types of susceptible individuals.

Stage of infection was modeled using the CD4-staging formalism developed by Cori et al. (2015). This includes four CD4-stage categories including AIDS, defined as patients with CD4 < 200. The staging system was modified to include a 1-year early infection (EHI) period instead of a 3-month acute infection period because previous work has shown the former system is more robust to phylodynamic estimation in the presence of phylogenetic error (Volz, et al. 2013a). The model allowed transmission rates to differ between EHI, chronic stages 2-4, and AIDS stage 5. This was parameterized with two parameters $\omega_{1}$, $\omega_{2}$ for the infectiousness of chronic and AIDS relative to EHI. Transmission rates were assumed to be lower in females than males by a factor of 75%.

Three risk groups were included in the models: heterosexual males (M), heterosexual females (F), and men who have sex with men (MSM). Transmissions were modeled between M and F, between MSM and other MSM, and between MSM and F. The transmission rates between MSM and F were controlled using two ‘assortativity’ parameters $p_{f}$ and $p_{\text{msm}}$. With probability $p_{\text{msm}}$ a transmission by a MSM was reserved for the same risk group, with the rest being allocated in proportion to susceptible population sizes of MSM and F. Similarly, a proportion $p_{f}$ of transmissions by F were reserved for M, with the rest being allocated in proportion to susceptible populations sizes of M and MSM. The transmission rates for MSM and the general population varied through time according to separate logistic functions, the parameters of which were estimated.

A summary of model parameters and priors are provided in Supplementary Table S1.

The model included diagnosis and treatment. The diagnosis rate was time-dependent and modeled using a logistic function, the parameters of which were estimated. The treatment rate depended on CD4-stage of infection based on prevailing guidelines in force in Nigeria which changed through time. Between 2004 and 2011, patients with CD4 < 200 were treated at a constant rate. After 2011, patients with CD4 < 300 were treated at a constant rate. Patients failed treatment at a fixed rate based on WHO surveillance (one per 4.02 person-years). Transmission rates were assumed to be reduced by 87% for patients on treatment.

Based on national census data, the population of the Federal Capital Territory of Nigeria, which includes Abuja and its metropolitan area was 1,405,201 in 2006. We modeled the population size of Abuja through time using an exponential function with growth rate based on census reports (2.53%/year). Phylodynamic estimation also required a compartment for the HIV infected population size outside of the Abuja region, as well as a specification of the rate that lineages are imported from outside of the Abuja region into the Abuja region. This compartment is termed the ‘Source’, since there is a source–sink relationship between the much larger global population of circulating HIV lineages and what was sampled in Abuja. We modeled the size of the source population using an exponential function (estimated rate ρ) and estimated one parameter for the importation rate of lineages from outside of Abuja (ψ).

The number of compartments for infected individuals was 45 = (five stages of infection) × (undiagnosed+ diagnosed+ treated) × (M + F+MSM). Additionally, there were three compartments for the number susceptible M, F, and MSM and a compartment for the size of the Source population. Results presented in the main text were based on a mathematical model with the following characteristic: An individual was characterized by

- stage of infection k∈(0–4), with 0 corresponding to EHI and 4 corresponding to AIDS
- risk group s∈(male, female, MSM)
- diagnosis and treatment status *d*∈(undiagnosed, diagnosed + not treated, diagnosed and treated)

The total rate of transmissions from all individuals in risk group s, stage k and diagnosis/treatment status d in an otherwise susceptible population is given by

$$\tau_{\text{skd}}(t)=f_{s}(t)\epsilon_{s}\omega_{k}\epsilon_{d}I_{\text{skd}}$$

where $I_{\text{skd}}$ is the number infected in that category, $\epsilon_{s}$ controls different baseline infectiousness for males, females, and MSM, $\epsilon_{d}$ may reduce infectiousness for diagnosed individuals and further reduce infectiousness of treated individuals. The function $f_{s}(t)$ describes secular trends in transmission rates through time using logistic functions. Separate logistic functions were used for MSM and the general population. Transmission rates were further scaled by a density dependent term $S_{s}/N_{s}$ for the corresponding recipients of transmission; for example, transmission from males to females is scaled by $S_{f}/N_{f}$. Transmissions between MSM and the GP are controlled by the $p_{f}$ and $p_{\text{msm}}$ parameters as follows:

- Transmission rate MSM→F:
  $$f_{\text{msm},k,d}^{F}(t)=\tau_{\text{msm},k,d}(t)(t-p_{\text{msm}})S_{F}/N_{F}$$
- Transmission rate MSM→MSM:
  $$f_{\text{msm},k,d}^{\text{msm}}(t)=\tau_{\text{msm},k,d}(t)(p_{\text{msm}}S_{\text{msm}}/N_{\text{msm}}+(1-p_{\text{msm}})(S_{\text{msm}}/(N_{f}+N_{\text{msm}})))$$
- Transmission rate F→MSM:
  $$f_{F,k,d}^{\text{msm}}(t)=\tau_{F,k,d}(t)(1-p_{f})S_{\text{msm}}/N_{\text{msm}}$$
- Transmission rate F→M:
  $$f_{F,k,d}^{M}(t)=\tau_{F,k,d}(t)((1-p_{f})(S_{M}/(N_{M}+N_{msm}))+p_{f}S_{M}/N_{M})$$

The model included non-adherence to treatment by including a rate for patients to revert from treated to diagnosed and untreated based on DHS surveillance, which reports 90% retention within one year of starting care. The per capita non-adherence rate was 24.8% per year. Stage progression for treated individuals was slowed to 25% of the baseline rate and was not estimated based on previous modeling work showing insensitivity of model fits to this parameter (Cori et al. 2014).

Multiple variations of this model were fitted and compared to this model:

- A model without diagnosis or treatment, and with a force of infection parameterized using exponential functions as in Granich et al. (2009) Several variations of the force of infection were tried involving polynomials up through order two. For example, with S susceptibles and I infected, the transmission rate may be modeled with the density dependent form $\beta SIe^{at+bI+c}/(S+I)$, where β,a,b,c are estimated. This approach was subsequently abandoned in favor of a logistic function with one fewer parameters. Two variations were tried with symmetric and asymmetric assortativity of females and MSM. Models with asymmetric assortativity were strongly supported.
- A model which allowed the proportion of men who are MSM to vary through time according to a linear function.
- A model with linear as opposed to logistic scaling of the force of infection with one fewer parameters.

The model was implemented in R and C ++ using the Rcpp package (Eddelbuettel et al. 2011), and source files are available at https://github.com/emvolz/nigeria_hiv_model, including examples of other model variations.

### 2.5 Phylodynamic analysis and model fitting

Structured coalescent models (Volz 2012) were used to infer demographic history and transmission rates in MSM and the general population. Recently, model-based phylogenetic inference has been used to characterize HIV transmission patterns by linking standard mathematical models for HIV epidemic dynamics with population genetic models. An advantage of model-based phylodynamic inference is that a wealth of non-genetic epidemiological surveillance data can be harnessed to calibrate mathematical models and refine parameter estimates. Furthermore, model development can draw on a wealth of expert knowledge that has developed over decades of modeling the HIV epidemic and statistical knowledge gained from fitting similar models to non-genetic surveillance data. Finally, the same models that are used for estimating HIV transmission patterns can be easily adapted for the purpose of predicting epidemic dynamics and to explore the likely impact and cost-effectiveness of different public health interventions.

Phylodynamic analysis was carried out using the *rcolgem* R package, (http://colgem.r-forge.r-project.org/) which was used to compute the likelihood of epidemiological parameters given time-scaled HIV phylogenies and the states of sampled individuals. The sample states were based on observed risk group (M, F, MSM, Source) as well as observed CD4 counts at the time of diagnosis where such data were available. Missing data were imputed on the basis of population frequencies of each CD4 stage at the time of sampling of each patient.

Fitting the epidemiological models made use of an extension to the general epidemiological structured coalescent model used in Volz (2012). This approach models the state of a lineage as a continuous time Markov process (CTMC) with time-dependent transition probabilities derived from the epidemiological model. The basic approach is described in Volz (2012), but we here provide examples of how the state of a lineage may change according to the model defined above. For example, given a lineage in a MSM in stage of infection *k* > 0, the lineage may revert to stage of infection *k* − 1 at a rate depending on the progression of disease. According to the coalescent model, this rate is $\gamma_{k-1}I_{\text{msm},k-1,d}/I_{\text{msm},k,d}$, where $\gamma_{k-1}$ is the rate of disease progression from stage k-1 to k. Similarly, transmission can change the state of a lineage. Suppose a lineage is in a MSM in the first stage of infection *k* = 0. The rate that this lineage will jump to a MSM host in stage and diagnosis category (*k*’,*d*’) depends on both the transmission rate from the donor deme and the number of infected MSM with EHI (stage zero) according to the following formula: $f_{\text{msm},k^{\prime },d^{\prime }}(t)/I_{\text{msm},0,d}(t)$.

Simulation priors were also used to constrain epidemic trajectories within plausible ranges based on WHO surveillance data. Prevalence of infection in the general population was constrained to fall within two standard errors of the estimated means of 0.035 in 2001 and 0.031 in 2012 based on WHO surveillance. Simulated trajectories were docked to the number of patients receiving ARV treatment in 2012 based on UNAIDS data: 491,021 in Nigeria, and estimated 4,706 in Abuja. Similarly, testing rates from simulations were docked to UNAIDS data for 2008 (6.6% per susceptible person year). Diagnosis and treatment rates in MSM were constrained to lie within two standard errors of the estimated values from the Bridging Trust study in 2012 (33% diagnosed, 31% of diagnosed were on treatment). Prevalence of infection in MSM at the end of 2010 and 2014 was constrained to lie within two standard errors of values estimated in the Bridging Trust study (34.9% and 44.5%). Incidence of infection in MSM was constrained to lie within two standard errors of values estimated in the Bridging Trust study in 2014 (13.9% per susceptible person year).

Likelihoods of epidemiological parameters were computed using a random sample of 20 phylogenies from the BEAST posterior. The likelihoods were based on combined G and CRF02_AG trees, since both clades have grown in tandem and viral lineages from both clades are in competition for susceptibles. Because coalescent analyses are based on a small sample of trees, we ensured that the particular selection of 20 trees was representative of the posterior by comparing the distribution of distances among and between trees using the metric developed by Kendall and Colijn (Kendall and Colijn, 2016) (Supplementary Material).

Models were fitted using a recently developed parallel Bayesian Metropolis-Hastings (pBMH) algorithm (Calderhead, 2014). This algorithm is a modification of traditional MH which makes multiple proposals in parallel and computes the likelihood of each in parallel. Fitting infectious disease models is challenging when the models are complex and likelihood calculations are slow. This approach has the advantage of efficiently utilizing high performance computing clusters when likelihood computations are slow. When computing the likelihood of a new proposal, a phylogeny was sampled uniformly at random from the posterior distribution estimated in BEAST. A Monte Carlo within Metropolis (MCWM) algorithm was used to compute the acceptance probability for each proposal. The pBMH algorithm utilized multivariate normal proposal distributions with variance equal to 0.1% the variance of the prior distributions.

In order to reduce the amount of computation required to fit each model, the dimension of the compartmental model was reduced after simulating each trajectory and prior to computing likelihoods with the *rcolgem* package. This was accomplished by combining chronic stages 2–4, and by combining diagnosed and treated compartments. This reduced the dimension of the system from 46 compartments to 19.

Model fitting made use of a computing cluster with 64 cores and more than 30,000 iterations for a total of more than 2 million likelihood calculations. The posterior distribution contained more than 40 thousand unique parameter sets.

Model variations were compared heuristically in terms of posterior likelihood and agreement with surveillance data.

To assess goodness of fit, we carried out a posterior predictive model simulation procedure (see Supplementary Material and Supplementary Fig. S3). Simulated lineages through time were compared to estimated lineages through time, showing good agreement.

### 2.6 Modeling test-and-treat interventions

To evaluate the impact of targeting interventions on key populations such as MSM, we simulated the fitted phylodynamic model for an extended time horizon 2016–2036. We modeled a five-year expansion (2016–2021) in diagnosis, treatment, and retention-in-care rates, such that by 2021 (1) 90% of people living with HIV are diagnosed, (2) 90% of treated individuals are retained in care and virally suppressed after one year of follow-up, and (3) 90% of diagnosed individuals receive ARV irrespective of CD4. We further compared scenarios such that the intervention was targeted to MSM only versus the entire population. Diagnosis, treatment, and retention-in-care rates increased linearly from the 2016 baseline (based on fitted models) to values giving the 90/90/90 diagnosis, treatment, and retention targets.

We simulated the fitted models for 2016–2036 and compared three scenarios:

1. No intervention—diagnosis, retention, and treatment rates remain at 2015 levels.
2. Intervention applied only to MSM.
3. Intervention applied to all people living with HIV.

Scenarios were compared in terms of infections averted and infections averted per person-year of treatment. Costs of the intervention were compared in terms of additional person-years of treatment and Infections averted per unit cost. Projected costs of treatment and new diagnoses in Nigeria were based recent estimates in Mitchell et al. (2015):

- $395 per person-year of treatment, which was an approximation obtained by taking an intermediate value between the higher cost for the first year of treatment ($515) and $365 subsequent years.
- $259 per diagnosis.

Supplementary Fig. S4 summarizes the impact and cost of the test-and-treat intervention by comparing targeted versus universal test-and-treat scenarios with a baseline case without any intervention.

## 3. Results

### 3.1 HIV incidence and prevalence patterns among MSM and other reproductive-aged populations

**Figure 2.**
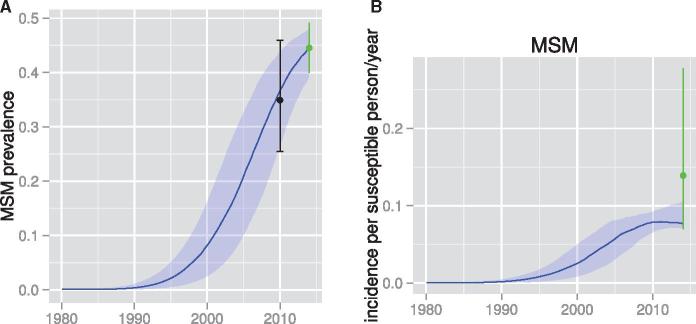
Estimated prevalence and incidence patterns through time based on phylodynamic analysis. Lines show median posterior estimates and shaded regions show 95% credible intervals. (A) Prevalence in MSM. The black error bar shows estimates from the Nigerian National IBBSS conducted in 2010 which were also used as priors in the phylodynamic analysis. The green error bar shows estimates based on the *TRUST* study conducted in 2014 which were also used as priors in the phylodynamic analysis. (B) Estimated incidence rate in MSM. The green error bar was estimated in 2014 from the *TRUST* study and was also used to define priors in the phylodynamic analysis.

There is currently no reliable incidence estimate in Nigeria or its trend over time. We applied phylodynamic analysis to estimate incidence rates for the MSM and reproductive-aged populations as shown in Fig. 2 and Supplementary Fig. S5. The incidence rate continues to increase among MSM from the 1980s to 2014 in contrast to the reproductive-aged population where we estimate that incidence is in decline. In 2014, the phylodynamic analysis yields estimates of incidence in MSM of 7.9% per susceptible person-years (95%CI, 7.0–10.4). The phylodynamic estimates substantially refine imprecise estimates obtained directly from follow-up in the *TRUST* Cohort (green error bar) with incidence rate 13.9 per 100 person-years (95%CI, 7.0–27.8).

The estimated prevalence substantially refines priors based on the HIV Integrated Biological and Behavioral Surveillance (FMOH, 2010) conducted in 2010 and the observed *TRUST* Cohort estimate in 2014, 44% (green error bars in Fig. 2; 95%CI, 39–48%).

### 3.2 HIV transmission patterns between MSM and other reproductive-aged populations

**Figure 3.**
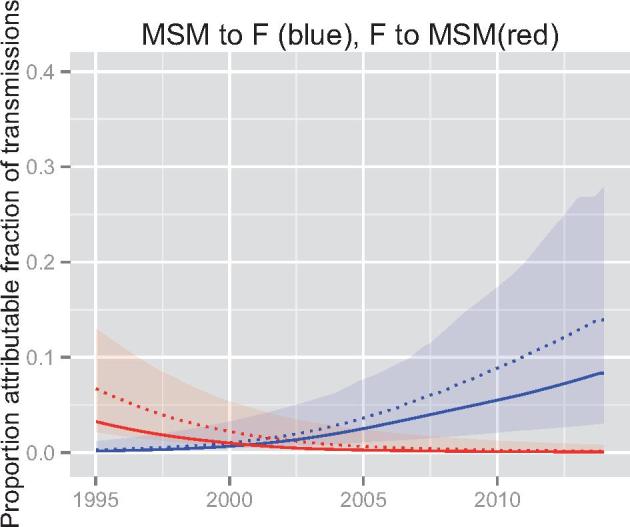
Proportion attributable fraction of transmissions based on phylodynamic analysis. Lines show median posterior estimates and shaded regions show the 95% credible interval. Blue denotes the proportion of infections among women in the general population due to the role of MSM. Solid line represents the maximum posterior estimate, and dashed represents the median posterior estimate. Red denotes the proportion of infections among MSM attributable to the role of the general population.

The phylodynamic analysis revealed strong evidence for asymmetric transmission rates between MSM and reproductive-aged females. In 2014, we estimate that 9.1% (95%CI, 3.8–18.6) of new infections in females are attributable to epidemiological interactions with the MSM group (Blue line). In the same year, only 0.2% (95%CI, 0.06–0.3) of new infections in MSM are attributable to females (Red line). Figure 3 shows the estimated trends in the population attributable fraction of transmission between MSM and females in the general population.

### 3.3 Predicting impact of universal access to HIV treatment

With the World Health Organization (WHO) call on the need to re-energize HIV programs for key populations (WHO 2015) and recently revised guidelines on ART initiation at any CD4 cell count (World Health Organization 2016), we sought to evaluate the impact of a universal test-and-treat on the modeled HIV transmission dynamics. Universal coverage of ART (UCT) leads to a large reduction in cumulative infections; 26.6% (95% CI, 23.7–29.2%) of infections are averted over five years and 55.4% (95% CI, 53.4–59.2%) of infections are averted over 20 years. Specific treatment approaches for all MSM living with HIV averts approximately half as many infections over 20 years (27.4%, 95% CI, 11.5–43.9%), but at greatly reduced cost. We predict that over five years (2016–2021), the cost of averting a single infection with targeted test-and-treat is $2,929 USD (95%CI, 2,229–3,737), and the same cost for universal test-and-treat is $7,569 USD (95%CI, 5,266–10,766). Over 20 years, the average cost per infection averted comes down to $1,662 USD (95% CI, 1017–2193) for targeted test-and-treat, but is only modestly reduced for universal test-and-treat ($7,343 USD).

## 4. Discussion

Transmission patterns between different risk groups show the disproportionate epidemiologic role of smaller populations of people living with HIV with unmet treatment needs. In the context of marginalization of key populations enforced by stigmatizing laws (Same Sex Marriage Prohibition Act 2013), these findings provide a public health rationale for governments and health leaders to address the needs of key populations with comprehensive preventative services. Structured coalescent analyses indicates that a substantial proportion of HIV infections in the general female population are linked epidemiologically to the MSM to female bridge which is consistent with reported sexual behaviors and observed incidence trends.

The genetic data reveal strong population structure and assortativity within risk-behavior groups. These observations are highly robust to the particular statistical methods used (Supplementary Material). Few transmission clusters marked by small evolutionary distance were detected. Of twelve pairs from subtypes G and CRF02 with evolutionary distance less than 1%, eleven were observed between MSM, and one was observed between MSM and general population. Higher clustering within MSM likely reflects much higher sampling effort in that risk group, and such small numbers of clusters do not allow inference of relative transmission rates between risk groups. Many factors may confound the inference of transmission rates between risk groups, including unequal sampling in different risk groups and changing transmission rates and population sizes through time. The model-based phylodynamic inference is robust to these sources of bias (Beerli and Felsenstein 2001; De Maio et al. 2015), and also reveals many more features of the epidemic history, such as how transmission rates between MSM and reproductive-aged populations have changed over time.

Patterns of HIV incidence and prevalence in MSM and other reproductive-aged adults have changed substantially since 1990 in Nigeria, with morbidity in MSM growing dramatically in recent years. There is also not a simple source-sink relationship between MSM and the general population. These findings strongly point to a failure to increase uptake of treatment and prevention services which is further exacerbated by stigmatizing laws (Schwartz et al. 2015). In contrast to the general population, both incidence and prevalence are yet to peak in MSM.

Due to increasing incidence and prevalence trends in MSM, the predicted impact of targeted test-and-treat averts many more infections per unit cost than UCT. We previously demonstrated effective implementation of test-and-treat strategy (Charurat et al. 2015) with a program attrition of 10% among individuals engaged in HIV treatment. We show that the gains of this intervention are not limited to MSM, since chains of HIV transmission cross back and forth between the general population and high-risk groups. Based on the estimated transmission rates between risk groups, we have predicted that over twenty years, a targeted test-and-treat strategy would avert about half as many infections as universal test-and-treat, but we estimate that the cost of targeted test-and-treat to only be about 16% of the cost of universal test-and-treat over 20 years. Fear of criminal sanctions as well as of discriminatory or stigmatizing health service provision may preclude the level of ‘test and treat’ services assumed in our model. In cases such as this, comprehensive HIV services should be understood to include legal and other support to MSM organizations to strengthen their capacity to advocate for access to services and changes in discriminatory laws and policies and support for human rights organizations and other allies that can assist MSM organizations in that effort.

Our findings should be interpreted with several caveats: The structured coalescent methods are premised on simple random sampling of infected hosts, however MSM patients were enrolled using RDS which yields correlated samples. We examined the data for evidence of correlation between HIV phylogenies and RDS recruitment trees, and we did not find any significant relationships (Supplementary Material). Second, the phylodynamic estimates depend on the accuracy of time-scaled phylogenies estimated using Bayesian relaxed-clock methods, and will be susceptible to factors that would bias such phylogenies, such as recombination or heterotachy. We examined our sequences for evidence of recombination events, and removed any sequences with low quality or evidence of recombination. Third, estimates are based only on sequences of subtypes G and CRF02_AG, and if epidemic dynamics differ substantially for other subtypes, our results may lack representativeness. Fourth, the phylodynamic estimates rely on the approximation that times of common ancestry between lineages correspond to the time of transmission events between hosts, which does not account for within-host evolution of the virus (Volz et al. 2013; Romero-Severson et al. 2014). Finally, it is possible that our model-based estimates would be biased by unmodeled heterogeneities that may influence HIV genetic diversity. While we examine a large range of models and accounted for many variables that may influence transmission rates, it is possible that failure to account for detailed sexual network structure in the epidemiological models may bias results.

These analyses have demonstrated the great potential for phylodynamic surveillance of infectious diseases to elucidate hidden transmission patterns which cannot be ascertained from standard surveillance data. As the quality and quantity of HIV sequence data improves, these analyses may be extended to gain a more fine-grained picture of transmission patterns and sexual network structure, as well as the role of other Key Populations and transmission hotspots to provide reliable estimates to guide high impact treatment and prevention interventions.

## Funding

The research reported in this publication was supported by the Global Fund to Fight AIDS, Tuberculosis and Malaria (GFATM, NGA-H-NACA). The *TRUST* Cohort is supported by U.S National Institutes of Health under award numbers R01MH099001 and R01AI120913 and by a cooperative agreement (W81XWH-11-2-0174) between the Henry M. Jackson Foundation for the Advancement of Military Medicine, Inc., and the U.S. Department of Defense. The *NeuroAIDS* Cohort is supported by the U.S. NIH award number R01MH086356. The *ACTION* Cohort is supported by the HHS/Centers for Disease Control and Prevention (CDC), Global AIDS Program with IHVN (U2GH000925). The content is solely the responsibility of the authors and does not necessarily represent the official views of the GFATM, the U.S. NIH, the U.S. Army, the Department of Defense, or other funders.

## Author Contributions

EV, WB, SB, and MC designed the study. NN, RN, PD, WR, and MC collected and managed data. EV, NN, and GK conceived the analyses. EV, WB, and MC drafted the manuscript and JI, SB, and MD provided critical review and editing. All authors have seen and approved the paper.

## Data Availability

NeuroAIDS study nucleotide sequences have been deposited with accession numbers: KY989243–KY989389. TDF study sequence accession numbers HQ843507-HQ843681. TRUST/Building study: KY989390–KY989540. Limited anonymized patient meta-data may be available from the authors upon request and conditional upon ethics approval.

## Supplementary data

Supplementary data are available at *Virus Evolution* online.

**Conflict of interest:** None declared.

## Supplementary Material

Supplementary Figure1

Supplementary Data
