# Supplementary material for: Phylodynamic analysis to inform prevention efforts in mixed HIV epidemics
Source: Virus Evol. 2017 Jul 28;3(2):vex014. doi: 10.1093/ve/vex014 (PMC5534066; doi:10.1093/ve/vex014)
Supplement: Supplementary Data [file vex014_supp.docx]

**Phylodynamic Analysis to Inform Prevention Efforts in Mixed HIV Epidemics: Supporting Materials and Methods**

**Authors:** Erik M. Volz PhD^1^*, Nicaise Ndembi PhD^2^, Rebecca Nowak PhD^3^, Gustavo H. Kijak PharmD PhD^4^ , John Idoko MBBS^5^, Patrick Dakum MBBS^2,3^, Walter Royal MD^3^, Stefan Baral MD^6^, Mark Dybul MD^7^, William A. Blattner MD^3^, Man Charurat PhD^3^

**Affiliations:**

^1^Department of Infectious Disease Epidemiology, Imperial College, London, UK

^2^Institute of Human Virology Nigeria, Abuja, Nigeria

^3^Institute of Human Virology, University of Maryland School of Medicine, Baltimore, Maryland, USA

^4.^U.S. Military HIV Research Program/Henry M. Jackson Foundation for the Advancement of Military Medicine, Bethesda, Maryland, USA

^5^National Agency for Control of AIDS, Abuja, Nigeria

^6^Center for Public Health and Human Rights, Johns Hopkins University, Baltimore, Maryland, USA

^7^Global Fund to Fight AIDS, Tuberculosis and Malaria, Geneva, Switzerland.

**Pol Sequencing**

To prepare the samples for genetic analysis, 500 µl of plasma were subjected to low-speed centrifugation to remove debris. The virus is then pelleted by centrifugation at 23,500 x g for 1 hr. The viral RNA was extracted using the QIAamp Viral RNA Mini Assay (QIAgen, Valencia, CA) and stored at –80^o^C. Reverse transcription–polymerase chain reaction (RT-PCR) was performed using the One Step Qiagen RT PCR Kit (Qiagen, Valencia, CA) according to the manufacturer’s protocol. The amplicon encoding protease and the amino terminus of RT (‘proRT’) were PCR amplified from RNA extracted using a nested strategy optimized for subtypes in Nigeria. In a total volume of 50 μl, the first round reaction was use PolF1yn (5’ –CAYTAGARGAAA TGATGACAGCAT– 3’) and PolR2yn (5’ –TCTGCCARTTCYARTTCTGC– 3’) followed by Pro5Fyn (5’ ARAAATTGCARGGCCCC TAGRAA– 3’) and ProRTyn (5’ -TTTTCCCAC TAAYTTCTGTATRTCATTG ACA– 3’) for the second round. Cycling conditions for the first round began with a hot start at 95^o^C for 10 minutes (m) then 45 cycles of (94^o^C, 30 seconds (s); 55^o^C, 30 s; 72^o^C 1.5 m), followed by 72^o^C for 7 m. The template for the second round was 1 μl from the first round and the cycling conditions was the same except that the number of cycles was 30 instead of 45. Amplification products were sequenced with Applied Biosystems 3130 automated sequencer (Applied Biosystems, Foster City, CA).

**Sequence quality control**

Obtained sequencing electropherograms were visually inspected using Sequencher 5.4 (Gene Codes Corp., Ann Arbor, Michigan, USA) at two independent laboratories (the Institute of Human Virology Nigeria, Abuja, Nigeria and the U.S. Military HIV Research Program in Bethesda, Maryland, USA) to verify that each nucleotide base was covered at least by 3 reads, one of which had to be in the opposite direction as the other two. Sequences were first aligned using HIVAlign (<http://www.hiv.lanl.gov/content/sequence/VIRALIGN/viralign.html>) and the alignments were manually edited using Geneious ([http://www.geneious.com](http://www.geneious.com/))[[1]](https://paperpile.com/c/9XIllA/JpMO). Sequence genetic relatedness was assessed in MEGA version 5.2.2. Samples whose sequences were <1.0% different and had been processed on the same day were re-processed and re-sequenced to rule out cross-sample contamination.

**HIV-1 subtype distribution based on HIV-1 pol analysis**

Central and West Africa is home to the highest level of HIV-1 genetic diversity worldwide presenting a major obstacle for the study of molecular epidemiology[[2]](https://paperpile.com/c/9XIllA/y04I). We conducted *pol* sequencing on 659 samples collected from the four population-based studies described above (Figure 1 main text). As expected, the main clades represented in these cohorts were subtype G and CRF02_AG. Other clades were also noted (e.g., subtypes A1, C, D, F1, K, CRF01_AE, CRF11_cpx, CRF18_cpx, CRF22_01A1) (data not shown). Numerous sequences were unique recombinants among all of these clades. With some notable exceptions, sequences from different cohorts appeared intermingled in the phylogeny.

**Subtype representation in different populations**

Overall, there was a balanced representation of subtype G and CRF02_AG (Figure 1, main text). In the *ACTION*, *NeuroAIDS* and *TDF* cohorts, subtype G was at 43-48% and CRF02_AG was at 28-44%, while the *TRUST Cohort* presented a relatively higher proportion of CRF02_AG (54%) at the expense of subtype G (22%, p<0.0001). Notably, previous characterizations of proviral sequencing from acutely HIV-infected high-risk Nigerian populations identified higher CRF02_AG compared to subtype G[[3]](https://paperpile.com/c/9XIllA/X3wn).

**HIV prevalence patterns among MSM and other reproductive-aged populations**

Nigeria, which reported its first two HIV cases in 1986, has recorded population-based HIV prevalence estimates increasing from 1.8% (1991) to 5.8% (2001). Thereafter, the prevalence declined over the years to 4.6% in 2008 and 4.1% in 2010. The majority of these data were obtained from the early Sero-prevalence Sentinel Survey Among the Antenatal Clinic Attendees[[4,5]](https://paperpile.com/c/9XIllA/xCyU+mtdT) and the National HIV/AIDS Reproductive Health Survey[[6]](https://paperpile.com/c/9XIllA/0URP). Estimated prevalence from 1980 to 2014 based on phylodynamic analysis find greater prevalence among MSM which is yet to peak in Nigeria contrary to patterns observed in the general population.

**Sample design and phylodynamic inference**

The phylodynamic inference methods used in this study are based on the assumption of simple random sampling within each deme (category of infected host)[[7]](https://paperpile.com/c/9XIllA/KyTD), and failure to account for non-random sampling of study participants may bias phylodynamic estimates. Participants in the Bridging Trust were sampled using Respondent Driven Sampling(RDS), a chain referral sample design which is known to produce highly correlated samples[[8,9]](https://paperpile.com/c/9XIllA/pIrJ+fFsS). In chain referral designs, sample units recruit one another into the study by drawing on their own network of friends and sexual relations. A recruiter is often observed to be more similar to a recruit due to like-with-like assortative mixing in social networks. This means that sample units collected by RDS can not be considered independent random draws from a sampling frame; rather sample units will be correlated, and correlation will decrease to zero with increasing distance within a recruitment chain.

We can inspect the influence of the RDS design on phylodynamic inference by characterizing the cross-correlation between HIV phylogenies and the RDS recruitment trees. We computed the distance between all pairs of sample units within RDS recruitment trees (the number of intermediate referrals between two participants within a recruitment tree). Distances between pairs of participants who were in different recruitment trees were treated as missing data. We also computed the temporal distance between all pairs of HIV sequences. This was based on a random sample of 20 phylogenies from the BEAST posterior distribution. RDS and phylogenetic distances were standardized (Z-scores), and then analyzed as follows:

• Distances were weakly correlated: Pearson
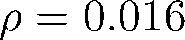


• A linear regression did not show significant relationship between distances (
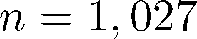
, 2-sided
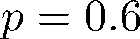
)

• To investigate the hypothesis that RDS recruiter-recruit pairs are more likely to be transmission pairs, we fit a logistic regression of a variable representing direct recruitment between pairs (RDS distance = 1) on standardized phylogenetic distances. This did not show a significant relationship (2 sided
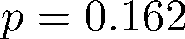
)

Although low correlation between RDS recruitment trees and HIV phylogenies does not guarantee that the RDS design did not bias results, it provides reassurance that the study is not biased by oversampling of transmission pairs.

**Characterizing HIV lineage migration with clustering, parsimony, and discrete trait analysis**

In addition to the phylodynamic modeling approach, we estimated migration rates of HIV lineages between risk groups based on conventional parsimony and maximum likelihood approaches.

• We tabulated the number of closely related pairs of sequences using a 1.5% evolutionary distance threshold. Distances were computed using a TN93 substitution model, Gamma rate variation (
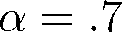
), based on BEAST analysis), and pairwise deletion of missing nucleotides.

• We used Fitch parsimony[[10]](https://paperpile.com/c/9XIllA/m9Zr) to infer ancestral states and the maximum parsimony number of jumps between demes.

• We conducted a discrete trait analysis (DTA), also termed a ‘mugration’ analysis, to estimate migration rates between demes. This analysis used the ‘ace’ function in the R package *ape* based on the algorithm in[[11]](https://paperpile.com/c/9XIllA/Qymc). Sample units were categorised into three demes: MSM, general population, and the global reference samples which we term the ‘Source’ population.

For parsimony and DTA, results were computed and averaged for a random sample of 20 trees from the BEAST posterior distribution.

There are many aspects of the epidemiological process that these methods do not account for. For example, the DTA model is based on a time-invariant matrix of migration rates between demes, and does not account for nonlinear changes in incidence and prevalence in different risk groups, and has recently been shown to not be robust to unequal sampling between demes[[12]](https://paperpile.com/c/9XIllA/UGzx). In our study, sampling was quite skewed, with a relatively large proportion of MSM sampled and a much smaller proportion of sequences from the general population. Despite these drawbacks, results from simple parsimony and DTA analyses qualitatively agree with our main findings.

Table S2 shows the matrix of migration rate estimated by DTA. Migration from the global source population to the Abuja demes (GP and MSM) greatly exceeds migration from Abuja to the source population. Migration from the GP deme to MSM is greater by 36% to migration from MSM to GP. Note also that the MSM deme is much smaller. The migration rates MSM
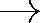
GP were not significantly different from the migration rates GP
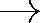
MSM (
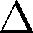
log likelihood = 0.11 relative to symmetric rates model).

Table S3 shows the number of HIV lineage jumps within and between demes inferred by maximum parsimony. Many more jumps are inferred within MSM and within GP demes than between GP and MSM, which reflects strong population structure and assortative mixing of the different risk groups. More jumps are observed from GP to MSM than vice versa, and this difference is significant (t test
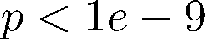
). Note that this difference is expected given the much larger size of GP, and the large number of jumps from MSM to GP is striking in light of the large disparity of deme sizes.

Finally, we summarize the number of clusters (pairs of closely related sequences) within and between risk groups with a 1.5% genetic distance. We find twelve clusters. There is only one cluster which spans the MSM and GP groups, and it is not possible to ascertain lineage migration rates between risk groups on the basis of clusters alone. Most clusters (11 of 12) are between MSM, which reflects the much higher sampling proportion within that group in the Trust study.

Migration rates between MSM and GP may have changed dramatically through time. The primary phylodynamic analysis described in the main text shows that similar proportion of transmissions flowed from GP to MSM as vice versa during the early epidemic, but this trend has reversed as prevalence and incidence in MSM has risen dramatically in recent years. Such trends can not be captured using these conventional DTA models with fixed migration rates.

**Posterior predictive model checking**

As a final check on goodness of fit, we conducted the following predictive simulations with comparisons to estimated phylogenies:

1. Ten parameter vectors
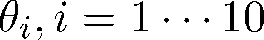
 were drawn from the estimated posterior distribution

2. Epidemic simulations were carried out for each
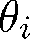
 yielding timeseries
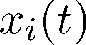
 (incidence and prevalence in each deme)

3. Structured coalescent simulations using the methods described in[[7]](https://paperpile.com/c/9XIllA/KyTD) were carried out based on output from each epidemic simulation, yielding coalescent trees
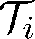


4. For each coalescent tree, the number of lineages as a function of time (NLFT) was computed for 100 time points distributed uniformly over the height of each tree

5. The empirical NLFT was computed based on a sample of twenty trees from the BEAST posterior distribution

Figure S3 compares the posterior simulated NLFT with the NLFT based on BEAST phylogenies. Note that because samples are collected heterochronously through time, the number of extant lineages may increase or decrease as one computes the number of lineages into the past. A comparison of residuals shows excellent agreement between simulations and phylogenies. Note that in general, the coalescent model may fit one of the posterior BEAST trees much better than the others, so we would not expect the variance in simulated NLFT to match the variance in BEAST NLFT, which is greater.

**Structured coalescent approximations**

Phylodynamic analyses made use of refinements to the structured coalescent model in [[7]](https://paperpile.com/c/9XIllA/KyTD)introduced in version 0.0.2 of the rcolgem R package (2014). These modifications addressed two issues:

1) Computational speed: Additional approximations are used to reduce the number of equations solved.

2) Reduce bias due to highly skewed coalescent rates within demes; the coalescent rate in some demes (such as MSM) may be higher than others, necessitating a refined model for how transition probabilities are computed.

We provide here a brief overview of the equations used to update lineage states in this version of the R package. Following the notation in[[7]](https://paperpile.com/c/9XIllA/KyTD),
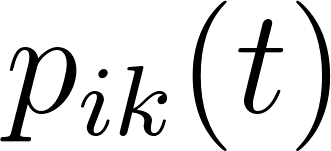
 represents the probability that lineage i is in state k at time t, and
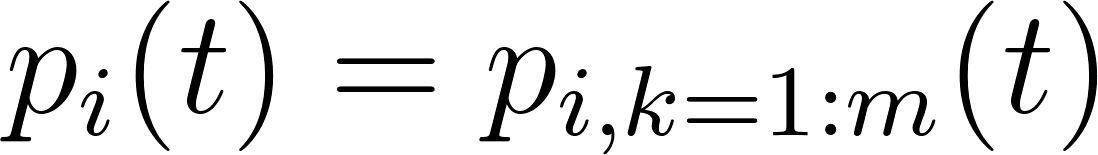
 is the vector of probabilities for lineage i with m demes. The coalescent model updates these states over the history of a tree, from the most recent sample to the root of the tree in the past. This is modeled as a continuous time Markov process. Over some interval of time
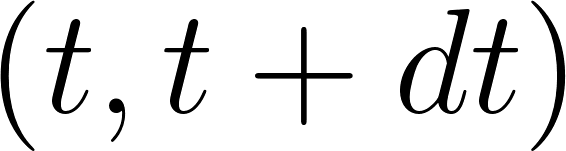
, we can update the states of lineages using a
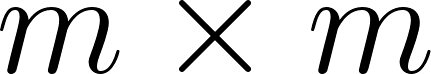
 matrix of transition probabilities Q:


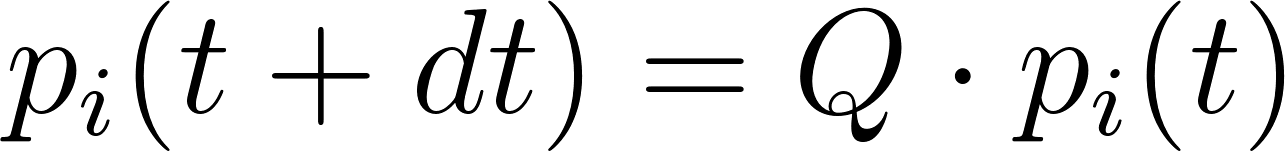


We solve
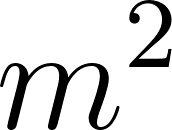
 equations to derive Q for each internode interval. Initialise
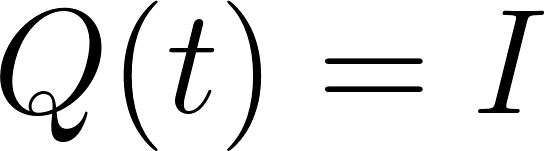
 to be the initial conditions for Q at the top of the internode interval (time t) where I is the identity matrix. Q is solved using the following system of ODEs:


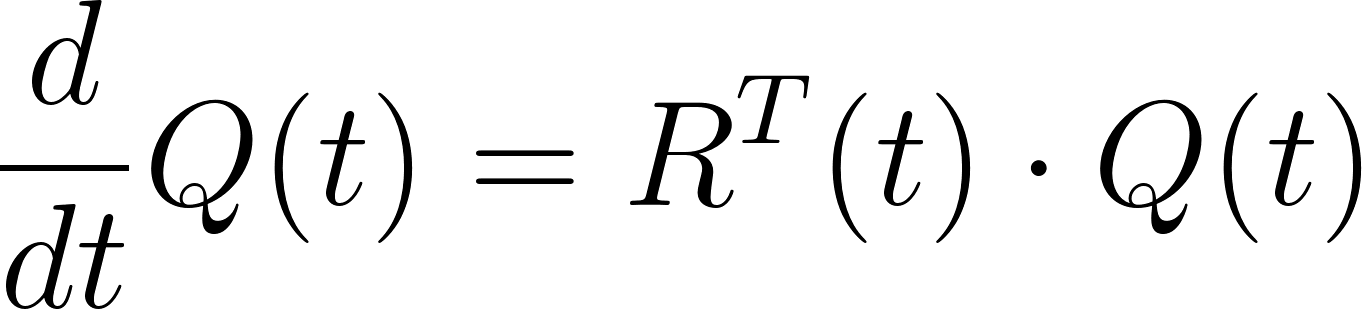


where R is the rate matrix with the following elements. In the following equations, refer to Volz 2012 for a definition of variables and for how these variables are derived from the epidemiological model.

R has the following elements:


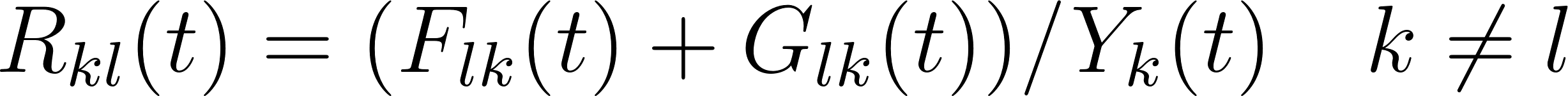


and on the diagonal:


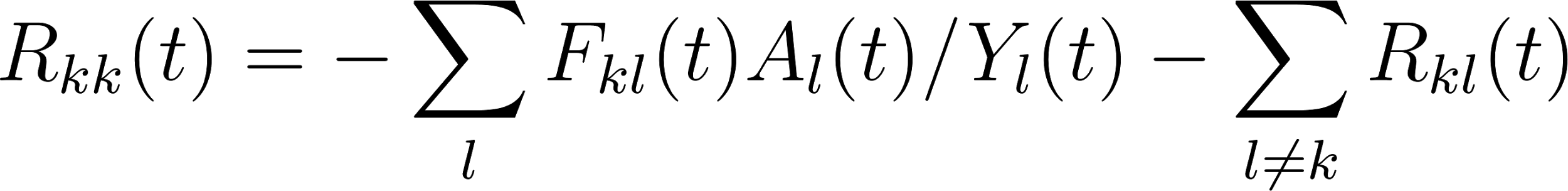


This model is highly similar to the one presented in [[7]](https://paperpile.com/c/9XIllA/KyTD) except for the inclusion of the term


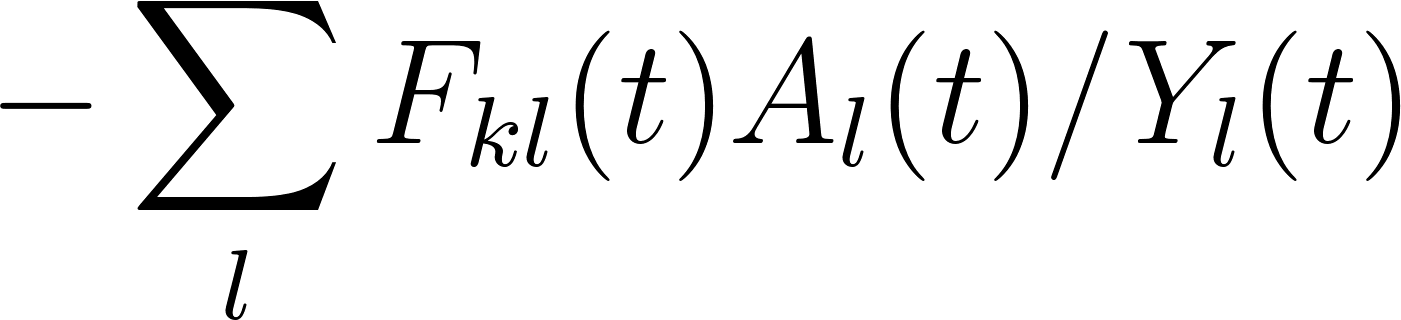


which adjusts for large disparities in coalescent rates between demes. At the bottom of the coalescent interval (at time t + dt), we renormalise the columns of Q to sum to one.

**Representativeness of posterior phylogeny sample**

Since only a sample of twenty phylogenies was used for structured coalescent analysis, we checked if this relatively small sample provided a good representation of the complete posterior of phylogenies estimated with BEAST. To do this, we computed a distance metric between all pairs of trees in posterior sample of 200 trees combined with the twenty trees used for coalescent analysis. Distance was computed using the metric of Kendall and Colijn [[13]](https://paperpile.com/c/9XIllA/7TRw). This metric provides a distance between phylogenies which accounts for differences in both topology and branch length (we used weight parameter lambda = .25). Distances were computed using the treescape R package.

Figure S6 shows a plot of the first two principal components of the tree distance metric for both the twenty phylogenies included in the analysis and the larger set of posterior trees. We also computed the distribution of distances to the centroid of this distribution. The twenty phylogenies were not significantly different from the larger set of posterior trees (KS test, p = 0.86). Both sets of distances had similar characteristics (median = 58410 versus 58840) and similar interquartile range.

**References**

1. [Kearse M, Moir R, Wilson A, Stones-Havas S, Cheung M, Sturrock S, et al. Geneious Basic: an integrated and extendable desktop software platform for the organization and analysis of sequence data. Bioinformatics. 2012;28: 1647–1649.](http://paperpile.com/b/9XIllA/JpMO)

2. [Kijak GH, McCutchan FE. HIV diversity, molecular epidemiology, and the role of recombination. Curr Infect Dis Rep. 2005;7: 480–488.](http://paperpile.com/b/9XIllA/y04I)

3. [Charurat M, Nasidi A, Delaney K, Saidu A, Croxton T, Mondal P, et al. Characterization of acute HIV-1 infection in high-risk Nigerian populations. J Infect Dis. 2012;205: 1239–1247.](http://paperpile.com/b/9XIllA/X3wn)

4. [FMOH. 2003 National HIV/Syphilis Seroprevalence Sentinel Survey. 2004.](http://paperpile.com/b/9XIllA/xCyU)

5. [FMOH. National HIV/Syphilis Seroprevalence Sentinel Survey. Federal Ministry of Health, Abuja, Nigeria; 2008.](http://paperpile.com/b/9XIllA/mtdT)

6. [FMOH. HIV/STI Integrated Biological and Behavioral Surveillance Survey. Nigerian Federal Ministry of Health, Abuja, Nigeria; 2010.](http://paperpile.com/b/9XIllA/0URP)

7. [Volz EM. Complex population dynamics and the coalescent under neutrality. Genetics. 2012;190: 187–201.](http://paperpile.com/b/9XIllA/KyTD)

8. [Salganik MJ, Heckathorn DD. Sampling and estimation in hidden populations using respondent-driven sampling. Sociol Methodol. Wiley Online Library; 2004;34: 193–240.](http://paperpile.com/b/9XIllA/pIrJ)

9. [Volz E, Heckathorn DD. Probability based estimation theory for respondent driven sampling. J Off Stat. Statistics Sweden (SCB); 2008;24: 79.](http://paperpile.com/b/9XIllA/fFsS)

10. [Fitch WM. On the Problem of Discovering the Most Parsimonious Tree. Am Nat. 1977;111: 223–257.](http://paperpile.com/b/9XIllA/m9Zr)

11. [Pagel M. Detecting Correlated Evolution on Phylogenies: A General Method for the Comparative Analysis of Discrete Characters. Proceedings of the Royal Society of London B: Biological Sciences. The Royal Society; 1994;255: 37–45.](http://paperpile.com/b/9XIllA/Qymc)

12. [De Maio N, Wu C-H, O’Reilly KM, Wilson D. New Routes to Phylogeography: A Bayesian Structured Coalescent Approximation. PLoS Genet. 2015;11: e1005421.](http://paperpile.com/b/9XIllA/UGzx)

13. [Kendall M, Colijn C. Mapping Phylogenetic Trees to Reveal Distinct Patterns of Evolution. Mol Biol Evol. 2016;33: 2735–2743.](http://paperpile.com/b/9XIllA/7TRw)


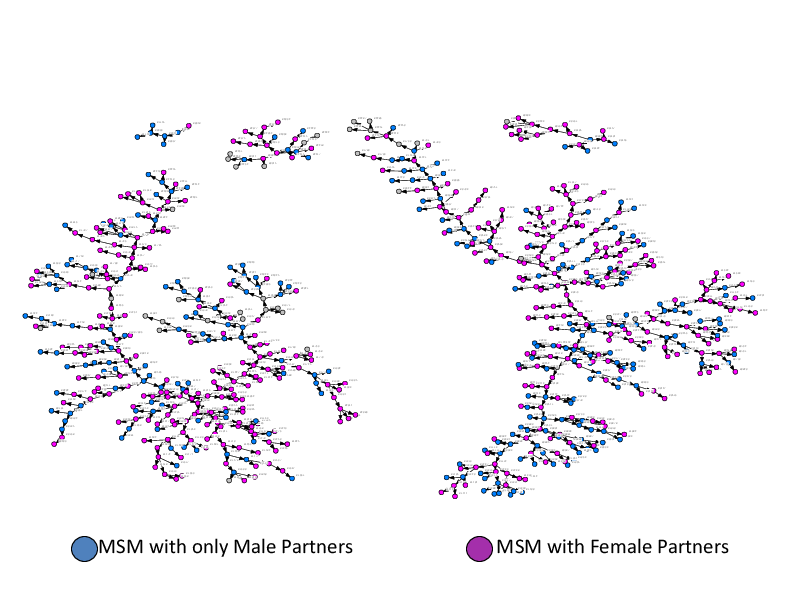


**Figure S1.** Respondent-driven sampling tree of men who have sex with men (MSM) in Abuja, Nigeria. Blue dots denote MSM with only male partners. Pink dots denote MSM with female partners. Arrows indicate the direction of recruitment into the study.

| 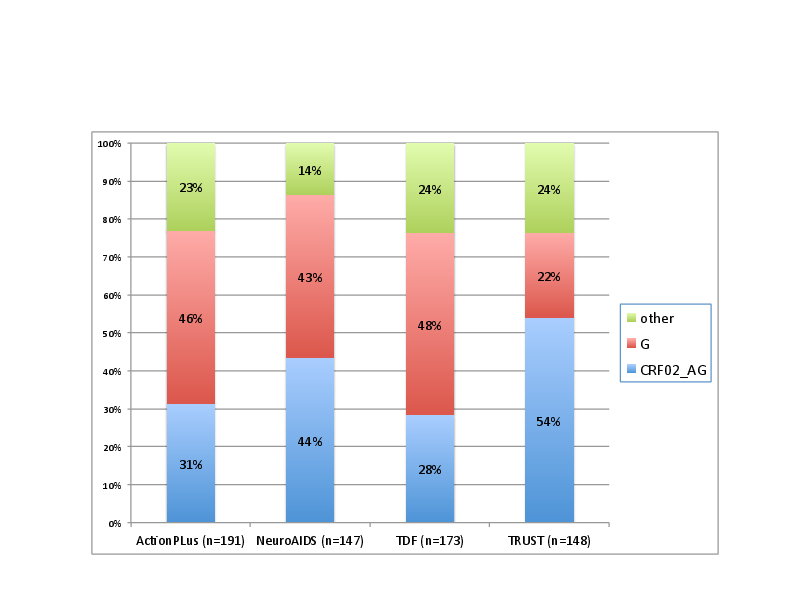 |
| --- |
| **Figure S2:** Frequency distribution of subtype G and CRF02_AG between different risk populations. The “Other” category includes other subtypes, recombinants between subtype G and CRF02_AE, and recombinants of subtype G and CRF02_AE with other clades. Each column corresponds to sequences from a different study (see main text). The TRUST study (last column) is the source of all sequences from men who have sex with men used in this study while other data sets were collected from the general population. |


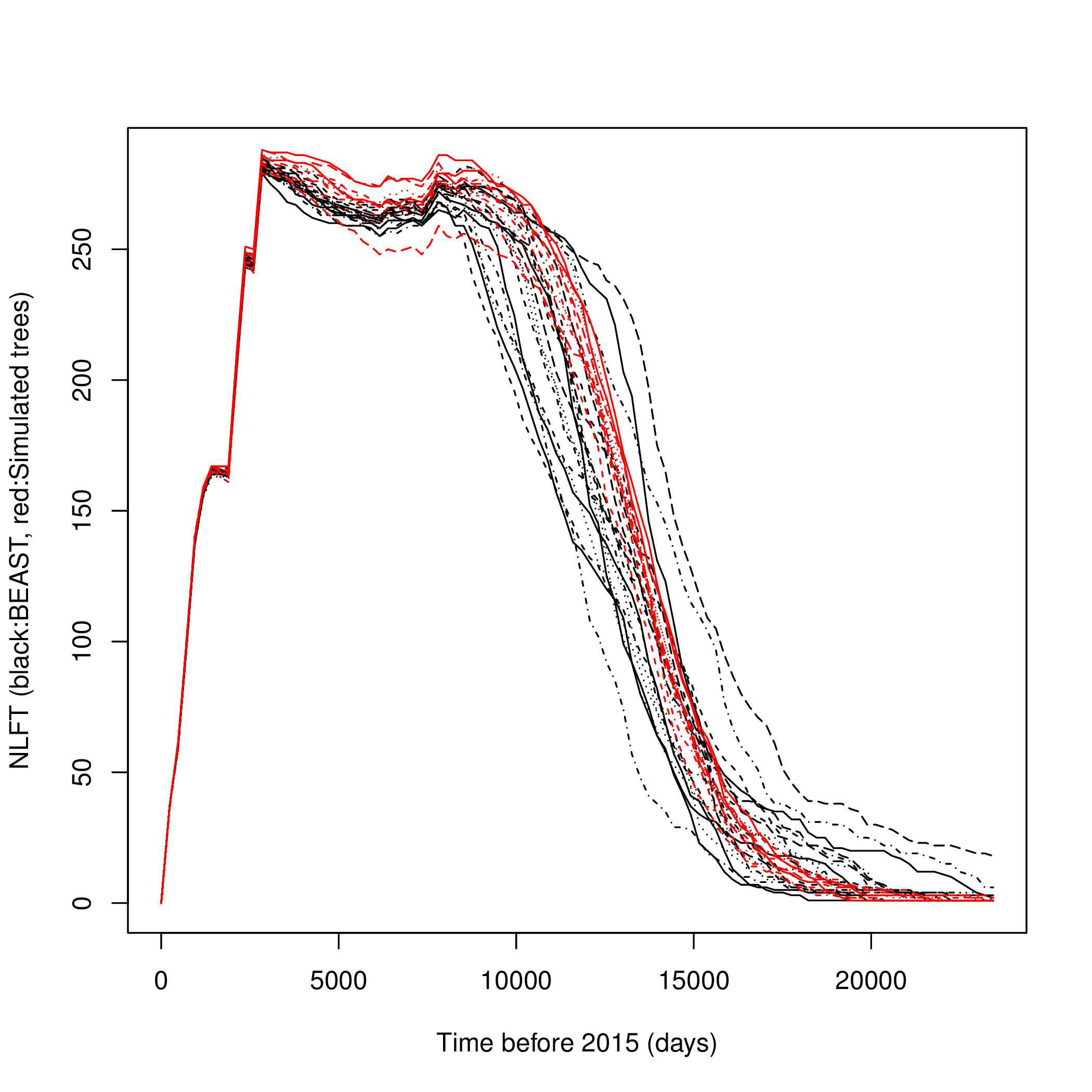


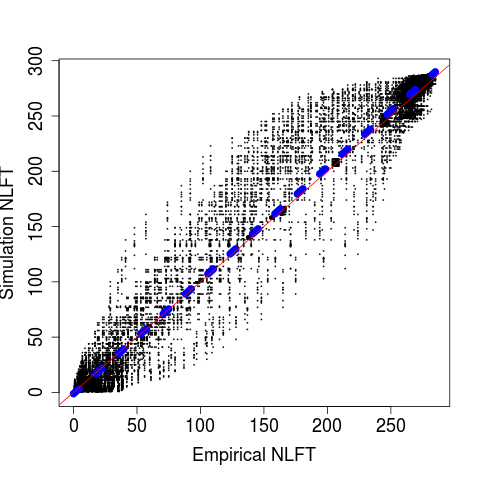


**Figure S3:** Top: The number of lineages as a function of time (NLFT) including predictions from posterior predictive simulation of coalescent trees (red) and samples from the BEAST posterior (black). Bottom: Simulated versus actual NLFT at 100 time points distributed uniformly over height of the trees. The red line shows the main diagonal and the dashed blue line shows a smoothed regression (loess) line.


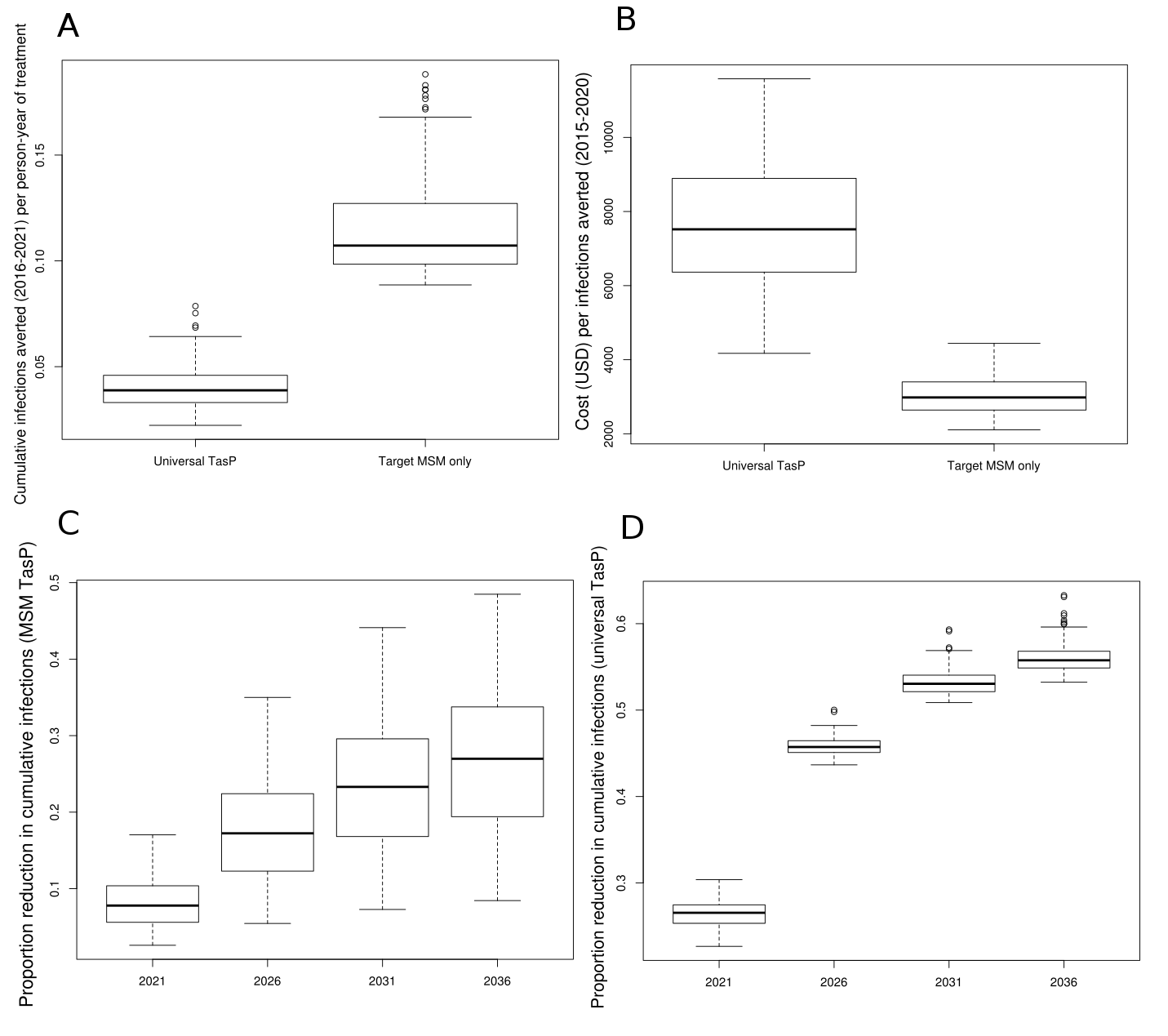
 **Figure S4:** Predicted effectiveness and cost of TasP interventions. A: The predicted proportion of infections averted per additional person-year of treatment. B: The cost (USD) of each additional infection averted relative to baseline with no intervention. C and D: Predicted proportion of infections averted over 20 year horizon if targeting MSM only (C) and the entire population (D) relative to baseline with no intervention. Error bars show 95% posterior predictive credible intervals and boxes show the posterior predictive interquartile range.


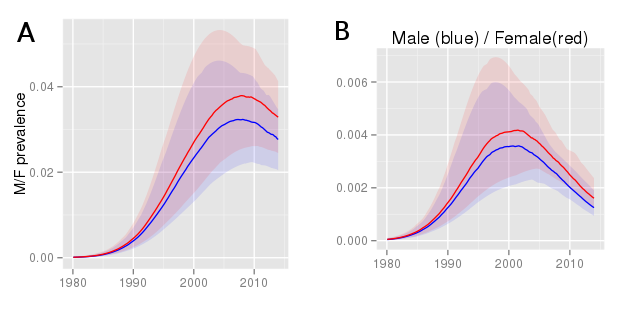


**Figure S5.** Estimated prevalence (A) and incidence (B) patterns through time based on phylodynamic analysis. Lines show median posterior estimates and shaded regions show 95% credible intervals. Blue and red lines indicate males and females respectively.


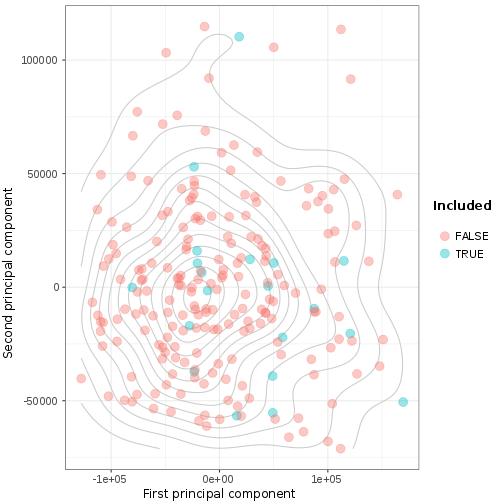


**Figure S6:** First two principal components of the distance matrix between posterior trees. Colours represent whether the phylogeny was included in the structured coalescent analysis. Contours show an estimated 2D Gaussian kernel density.

**Table S1: Epidemiological model parameter priors.**

| **Symbol** | **Definition** | **Prior** | **Units** |
| --- | --- | --- | --- |
| 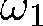 | Relative infectiousness chronic | Unif(0, .30 ) | dimensionless |
| 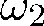 | Relative infectiousness AIDS | Unif(0, .30) | dimensionless |
| 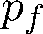 | Assortativity females | Beta(5,1) | dimensionless |
| 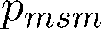 | Assortativity MSM | Beta(5,1) | dimensionless |
| 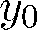 | Initial number infected | Unif(0,10) | Individuals |
| 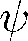 | Immigration rate | Unif(1/30yrs, 1/5yrs) | Per capita per year |
| 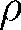 | Source growth rate | Unif(1/3.5yrs, 1/.8 yrs) | Per capita per year |
| 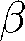 | Baseline transmission rate (M&F) | Lognorm(log(0.425/yr), .5 ) | Per capita per year |
| 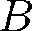 | Logistic rate parameter for M/F transmission | Lognorm( log(0.25), 1) | dimensionless |
| 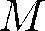 | Transmission rate M/F (logistic position) | Norm(1990, sd=5) | Year |
| 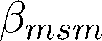 | Baseline transmission rate msm | Lognorm(log(0.425/yr), .15 ) | Per capita per year |
| 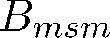 | Logistic rate for MSM transmission | Norm(.25, sd = .25) | dimensionless |
| 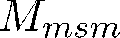 | Transmission rate msm (logistic position) | Norm(1990, sd = 6) | Year |
| 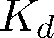 | Diagnosis rate (logistic max) | Lognorm(log(1/10yrs), 0.6) | Per capita per year |
| 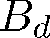 | Logistic rate parameter for diagnosis | Lognorm(log(0.5), 0.4) | dimensionless |
| 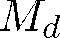 | Diagnosis rate (logistic position) | Norm(2006, sd = 1) | Year |
| 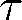 | Treatment rate | Lognorm(log(1/2yrs), 0.4) | Per capita per year |
| 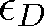 | Transmission reduction from diagnosis | Unif(0,1) | dimensionless |
| 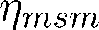 | proportion of males MSM | Unif(0.15%, 1% ) | dimensionless |

**Table S2:** ML estimates of migration rates (per lineage per year) using discrete trait analysis. Rates are computed between MSM, the general population (GP) and the ‘Source’ population, which represents global reference sequences. All rates are reported relative to the migration rate from MSM to GP (set to unity). Standard errors are reported parenthetically.

|  | **MSM** | **GP** | **Source** |
| --- | --- | --- | --- |
| **MSM** | NA | 1 (0.45) | 0.29 (0.50) |
| **GP** | 1.36(0.53) | NA | 0.25(0.35) |
| **Source** | 1.53(0.23) | 3.8(0.39) | NA |

**Table S3:** Number of jumps of HIV lineages within and between demes inferred by maximum parsimony.

|  | MSM | GP | Source |
| --- | --- | --- | --- |
| MSM | 54.55 | 5.7 | 3.85 |
| GP | 10.85 | 59.8 | 13.5 |
| Source | 12.6 | 25.4 | 128.55 |
