# Supplementary material for: Phylodynamic analysis to inform prevention efforts in mixed HIV epidemics
Source: Virus Evol. 2017 Jul 28;3(2):vex014. doi: 10.1093/ve/vex014 (PMC5534066; doi:10.1093/ve/vex014)
Supplement: Supplementary Figure1 [file vex014_supp_figure1.pdf]

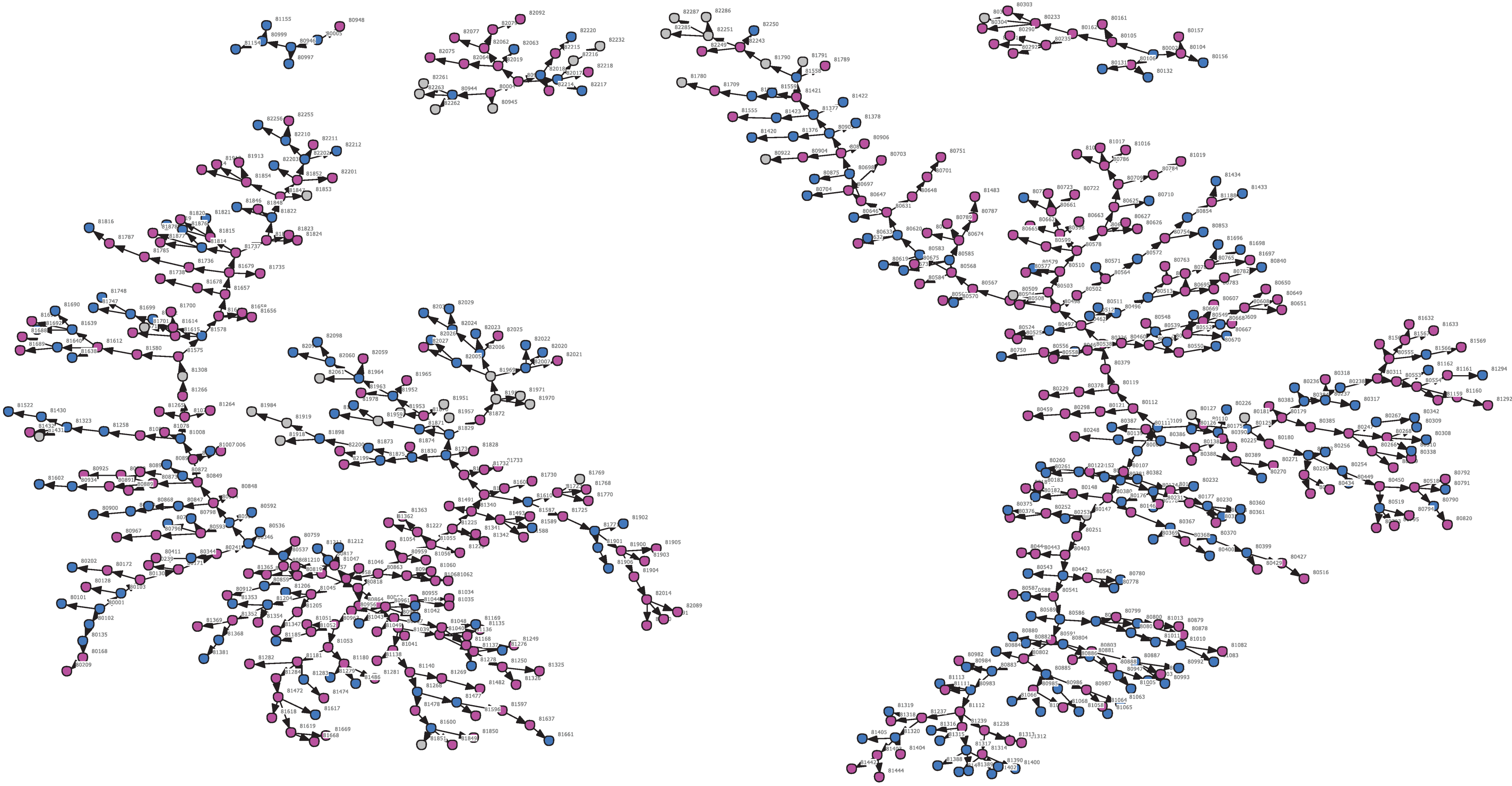

MSM with only Male Partners

MSM with Female Partners
